# Human Mesenchymal Stem Cell Derived Exosomes Enhance Cell‐Free Bone Regeneration by Altering Their miRNAs Profiles

**DOI:** 10.1002/advs.202001334

**Published:** 2020-08-07

**Authors:** Mengmeng Zhai, Ye Zhu, Mingying Yang, Chuanbin Mao

**Affiliations:** ^1^ Department of Chemistry and Biochemistry Stephenson Life Sciences Research Center University of Oklahoma Norman OK 73019 USA; ^2^ Institute of Applied Bioresource Research College of Animal Science Zhejiang University Hangzhou Zhejiang 310058 P. R. China; ^3^ School of Materials Science and Engineering Zhejiang University Hangzhou Zhejiang 310027 China

**Keywords:** bone regeneration, cell‐free scaffolds, exosomes, miRNAs

## Abstract

Implantation of stem cells for tissue regeneration faces significant challenges such as immune rejection and teratoma formation. Cell‐free tissue regeneration thus has a potential to avoid these problems. Stem cell derived exosomes do not cause immune rejection or generate malignant tumors. Here, exosomes that can induce osteogenic differentiation of human mesenchymal stem cells (hMSCs) are identified and used to decorate 3D‐printed titanium alloy scaffolds to achieve cell‐free bone regeneration. Specifically, the exosomes secreted by hMSCs osteogenically pre‐differentiated for different times are used to induce the osteogenesis of hMSCs. It is discovered that pre‐differentiation for 10 and 15 days leads to the production of osteogenic exosomes. The purified exosomes are then loaded into the scaffolds. It is found that the cell‐free exosome‐coated scaffolds regenerate bone tissue as efficiently as hMSC‐seeded exosome‐free scaffolds within 12 weeks. RNA‐sequencing suggests that the osteogenic exosomes induce the osteogenic differentiation by using their cargos, including upregulated osteogenic miRNAs (Hsa‐miR‐146a‐5p, Hsa‐miR‐503‐5p, Hsa‐miR‐483‐3p, and Hsa‐miR‐129‐5p) or downregulated anti‐osteogenic miRNAs (Hsa‐miR‐32‐5p, Hsa‐miR‐133a‐3p, and Hsa‐miR‐204‐5p), to activate the PI3K/Akt and MAPK signaling pathways. Consequently, identification of osteogenic exosomes secreted by pre‐differentiated stem cells and the use of them to replace stem cells represent a novel cell‐free bone regeneration strategy.

## Graphical abstract

Exosomes secreted from human mesenchymal stem cells (hMSCs) pre‐differentiated for a certain period of time can serve as inducers to induce osteogenic differentiation of hMSCs in vitro. They can decorate 3D printed titanium alloy scaffolds, which are further implanted into radial bone defect. They are found to enable the scaffolds to achieve efficient cell‐free bone regeneration in vivo.

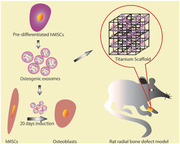

## 1 Introduction

Bone defects can be caused by various clinical diseases such as bone infections, bone tumor, skeletal abnormalities, congenital malformation, fractures, avascular necrosis, atrophic non‐unions, osteoporosis, and bone trauma.^[^ ^1^ ^]^ They are traditionally treated with autografts, allografts, and xenografts.^[^ ^2^ ^]^ However, these therapies have their own limitations. For the autografts, they are limited to donor sources and mismatched with the defect sites. For the allografts and xenografts, they have the risks of disease transmission and immune rejection.^[^ ^3^ ^]^ Meanwhile, the implantation of the stem cells, like the human mesenchymal stem cells (hMSCs)^[^ ^4^ ^]^ and human adipose‐derived stem cells,^[^ ^4^ ^]^ are considered an alternative strategy. However, implantation of stem cells faces significant challenges, including immune rejection,^[^ ^5^ ^]^ teratoma formation,^[^ ^6^, ^7^ ^]^ and undirected cell differentiation_._^[^ ^8^, ^9^ ^]^ When seeded with the stem cells before implantation, the 3D scaffolds may cause immune rejection,^[^ ^9^ ^]^ induce teratoma formation,^[^ ^10^ ^]^ and lead to undirected cell differentiation.^[^ ^11^ ^]^ Therefore, bone regeneration without the use of externally seeded stem cells, termed cell‐free regeneration, is a promising approach to solving these cell‐derived problems. Indeed, implantation of cell‐free scaffolds for cell‐free bone tissue regeneration^[^ ^12^ ^]^ has emerged as a promising strategy to avoid these problems.^[^ ^12^, ^13^, ^14^ ^]^

To achieve cell‐free tissue regeneration, the cell‐free 3D scaffolds should bear the biochemical and physical cues that can induce osteogenesis.^[^ ^15^ ^]^ One of the possible biochemical cues is the exosomes secreted by the cells, which are nanoparticles (30–200 nm) with lipid‐bilayers encapsulating signaling cargoes such as miRNAs.^[^ ^16^ ^]^ Stem cell‐derived exosomes are less immunogenic than the cells themselves because the exosomes contain a low amount of membrane proteins (e.g., major histocompatibility complex, MHC).^[^ ^17^ ^]^ The exosomes can keep their biological activities for a long time.^[^ ^18^ ^]^ Small soluble molecules in the exosomes, such as miRNAs, growth factors, cytokines, and transcription factors, are protected by the lipid bilayer and can be released to target tissues.^[^ ^19^ ^]^ Since the exosomes contain a range of genetic information,^[^ ^20^ ^]^ like miRNAs, they can direct immune modulation and cell‐to‐cell communication.^[^ ^21^ ^]^ As a result, when released from the cells, exosomes can have interactions with the recipient cells by adhering to their surface, and communicate with them by the lipid‐ligand receptor interactions, endocytic intake, or fusion with the cell membrane.^[^ ^22^ ^]^

Since MSCs will secret exosomes into the culture medium, MSC‐conditioned medium will contain exosomes. This might explain the reported findings that MSC‐conditioned medium could be used to promote tissue regeneration. For example, MSC‐conditioned medium could decrease the infarct size to treat myocardial ischemia.^[^ ^23^ ^]^ Such medium enhanced the migration and proliferation of kidney‐derived epithelial cells and increased the survival of the tubular cells in vivo.^[^ ^24^ ^]^ Intravenous injection of MSC‐derived exosomes promoted neurovascular remodeling.^[^ ^25^ ^]^ These findings indicate that the biochemical cues encapsulated in the exosomes might also induce the osteogenesis in vitro and in vivo.

**Scheme 1:**
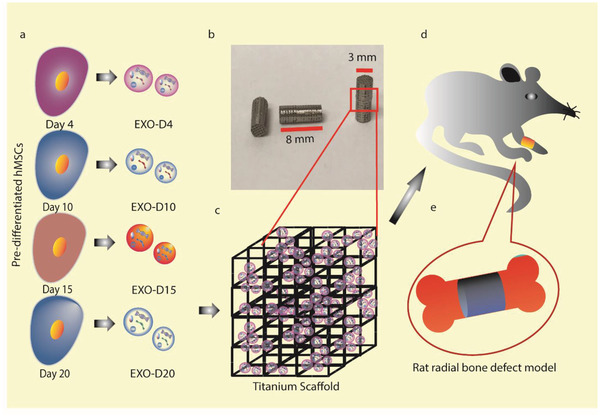
Cell‐free bone tissue regeneration by the stem cell derived exosomes. a) Exosomes were isolated from the pre‐differentiated hMSCs induced by the osteogenic medium for 4, 10, 15, 20 days, respectively. Osteogenic differentiation was tested to identify the exosomes that could induce the osteogenic differentiation of hMSCs. b) Representative structure and morphology of Ti‐scaffolds (8 mm in length and 3 mm in diameter). c–e) Osteogenic exosomes were seeded into the Ti‐scaffolds, which were then implanted into the rat radial bone defect model (d and e) for 4 and 12 weeks, respectively.

Hence, we identified osteogenic exosomes secreted from hMSCs and used the exosomes to decorate 3D printed titanium alloy scaffolds (Ti‐scaffolds) to achieve cell‐free bone regeneration (**Scheme** **1**). Specifically, we employed the exosomes derived from the hMSCs pre‐differentiated in osteogenic differentiation medium for 0, 4, 10, 15, and 20 days (termed EXO‐D0, EXO‐D4, EXO‐D10, EXO‐D15, and EXO‐D20, respectively) to identify the exosomes that can induce the osteogenic differentiation of hMSCs into osteoblasts in vitro. Then we filled cell‐free 3D printed Ti‐scaffolds with the osteogenic exosomes to achieve cell‐free bone regeneration. Ti‐scaffolds were used to support the exosomes because Ti is biocompatible, does not elicit immune reaction with the tissue,^[^ ^26^, ^27^ ^]^ and supports the attachment of bone cells and the mineralized bone matrix without any interposition.^[^ ^26^, ^28^ ^]^ In addition, Ti‐scaffolds have desired properties, such as the uniform structure, strength, low stiffness, high porosity, corrosion resistance, and high coefficient friction.^[^ ^29^ ^]^

We used real‐time PCR, immunofluorescence staining, Alizarin Red Staining, and alkaline phosphatase (ALP) activity to confirm the osteogenesis of the hMSCs under the induction of hMSC‐derived exosomes. The cell‐free 3D Ti‐scaffolds were also applied to induce bone tissue regeneration for 4 and 12 weeks in vivo. We found that the exosomes (EXO‐D10 and EXO‐D15) carried the differentiation‐inducing miRNAs (like Hsa‐miR‐146a‐5p, Hsa‐miR‐503‐5p, Hsa‐miR‐483‐3p, Hsa‐miR‐129‐5p, Hsa‐miR‐32‐5p, Hsa‐miR‐133a‐3p and Hsa‐miR‐204‐5p) and thus could induce the osteogenic differentiation of the hMSCs in vitro and enable the cell‐free Ti‐scaffolds to achieve bone tissue regeneration in vivo by activation of PI3K/Akt and MAPK signaling pathway. Thus, the exosomes from the osteogenically pre‐differentiated hMSCs are inducers for the cell‐free bone tissue regeneration.

## 2 Results

### 2.1 The Identification and Quantification of the Exosomes Derived from the hMSCs

**Figure 1:**
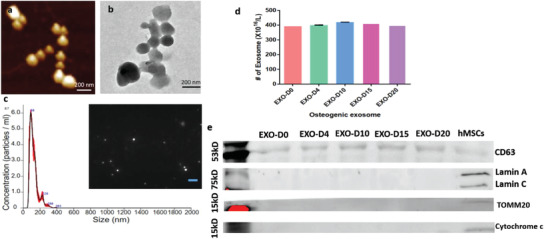
The characterization of the stem cell derived exosomes. a) AFM and b) TEM showing the size and morphology of the exosomes derived from hMSCs. Scale bar: 200 nm. c) The size and concentration of the hMSCs‐derived exosomes by the NanosightNS300. The inset is an image showing the snapshot of video tracking. Scale bar: 800 nm. d) The concentration of exosomes (derived from the pre‐differentiated hMSCs on day 0, day 4, day 10, day 15, and day 20, respectively) determined by EXOCET Exosome Quantitation kit. e) The Western blot analysis of the exosome derived from the pre‐differentiated hMSCs and hMSCs. 20 µg of the exosome proteins were loaded into the lane. Exosome specific anti‐CD63 primary antibody was used. Lamin A and Lamin C: Nuclear marker; TOMM20 and Cytochrome c: Mitochondrial marker.

The exosomes secreted by the pre‐differentiated hMSCs were found to be indeed nearly spherical nanoparticles around 143.0 ± 9.3 nm by the NanosightNS300 (**Figure** **1**c), consistent with the observations by atomic force microscopy (AFM) and transmission electron microscopy (TEM) (Figure 1a,b). Their concentrations were determined to be as high as ≈4.0 × 10^9^ particles per µL (Figure 1d) for EXO‐D0, EXO‐D4, EXO‐D10, EXO‐D15, and EXO‐D20. The exosomal protein marker CD63 was also detected by the western blot (Figure 1e). In addition, Western blot shows that the exosomes derived from hMSCs did not present the markers for cell nuclei and mitochondria (Figure 1e), further confirming the identity of the exosomes.

### 2.2 The Exosomes Derived from the Pre‐Differentiated Stem Cells Induce the Osteogenic Differentiation of hMSCs In Vitro

**Figure 2:**
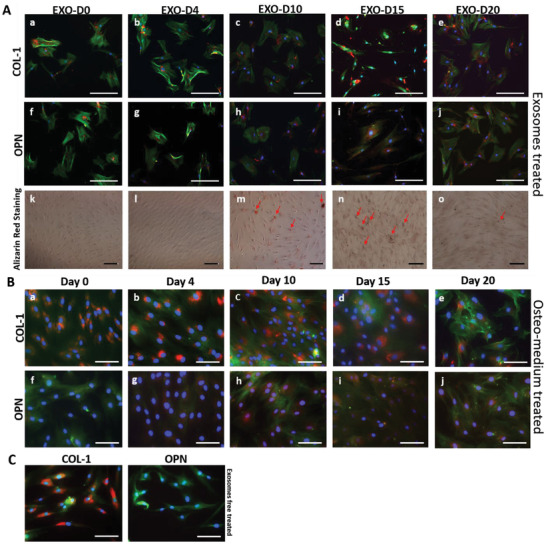
Osteogenic differentiation of hMSCs by the osteogenic exosomes. A,a–j) Immunofluorescence staining of the osteogenic markers (COL‐1 [a–e]; OPN [f–j]) in the hMSCs showing the osteogenic differentiation on day 20 induced by hMSCs‐derived osteogenic exosomes (EXO‐D0, EXO‐D4, EXO‐D10, EXO‐D15, and EXO‐D20) and B) the osteogenic medium. The red color of the cells ([h–j] in [A]) treated with the EXO‐D10, EXO‐D15, and EXO‐D20 is much deeper than that of the cells in (f) and (g). Similarly, the red color of the cells ([h–j] in [B]) treated with the osteogenic medium is much deeper than the other control cells in (f) and (g). Blue (DAPI), nuclei; green (FITC), F‐actin; red (TRITC), OPN and COL‐1. k–o) Bright field images of the Alizarin Red staining for the osteogenesis of hMSCs induced by the osteogenic exosomes. The red deposit is the calcium nodule and indicated by the arrows. Scale bar: 100 µm.

**Figure 3:**
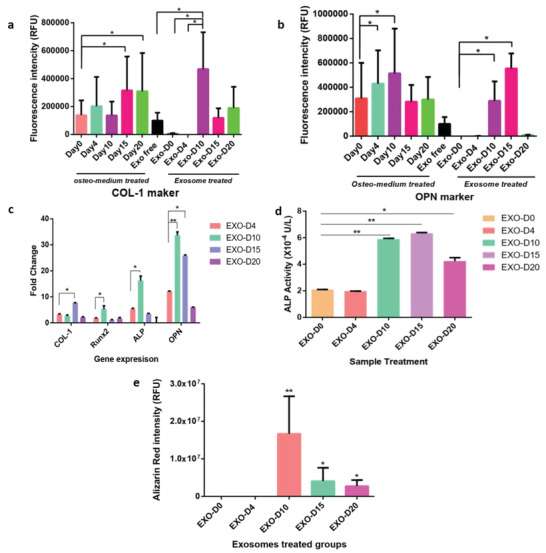
The quantitation of the immunofluorescence staining, gene expression and ALP activity. The relative fluorescence intensity (RFU) of the a) Col‐1 and b) OPN marker of the immunofluorescence staining. c) Real‐Time PCR analysis of the osteogenic markers (COL‐1, Runx2, ALP, and OPN) showing the osteogenesis of hMSCs induced by the osteogenic exosomes at the gene level. Each gene expression was compared with that of the EXO‐D4 treated group. d) ALP activity of osteogenesis of hMSCs induced by the osteogenic exosomes. Each ALP activity was compared with that of the EXO‐D0 treated group. e) The Alizarin Red intensity after all kinds of exosome induction. Each exosome treated group was compared with the EXO‐D0 treated group. *p < 0.05; **p < 0.01, *N* = 3.

To evaluate the osteogenic performance of the exosomes, we used the non‐osteogenic and osteogenic exosomes as an inducer to activate the osteogenesis of the hMSCs. The hMSCs were incubated with the EXO‐D0 (non‐osteogenic exosomes), EXO‐D4, EXO‐D10, EXO‐D15, EXO‐D20 (osteogenic exosomes) for 20 days. Immunofluorescence microscopy shows that the type 1 collagen (COL‐1) expression of the cells treated with different osteogenic exosomes (**Figure** **2**A, a–e) or with osteogenic medium (Figure 2B, a–e) is almost same because COL‐1 is not an osteo‐specific differentiation marker. Meanwhile, we also quantified the immunofluorescence intensity of the COL‐1 expression of the hMSCs induced by the osteogenic exosomes and medium. When the cells were induced by osteogenic medium, their COL‐1 immunofluorescence intensity on day 15 and day 20 is much higher than on the earlier day (**Figure** **3**a). When the cells were treated by exosomes, their COL‐1 immunofluorescence intensity for the EXO‐D15 group showed the highest signal among all groups (Figure 3a). However, there is no significant difference between osteogenic exosome treatment and osteogenic medium treatment. When the hMSCs were treated by different exosomes, the osteopontin (OPN) expression in Figure 2A, h–j is much higher than that in Figure 2A, f–g, indicating that the EXO‐D10, EXO‐D15, and EXO‐D20 have the higher differentiation‐inducing ability for the osteogenesis of the hMSCs. This trend is similar to the situation where the cells were treated by the osteogenic medium induced cells (Figure 2B, h–j). For the intensity of the immunofluorescence staining, we found that OPN expression of the cells for 4 days and 10 days showed a higher level than that for other time points when the cells were treated by osteogenic medium (Figure 3b). When the cells were treated by exosomes, the OPN expression of the cells treated by the EXO‐D10 and EXO‐D15 presented a higher level than that for the other exosome groups (Figure 3b). Similarly, no significant difference between the osteogenic exosome treatment and osteogenic medium treatment was found in OPN expression. To further confirm the exosome's osteogenic differentiation ability, we performed the real‐time PCR analysis of the osteo‐specific markers (Runx2, ALP, and OPN). Figure 3c shows that OPN gene expression of the cells treated with the EXO‐D10 is the highest, and OPN gene expression in the EXO‐D15 group is a little bit lower than that in the EXO‐D10 group but still higher than the other groups. For the ALP gene expression, the cells incubated with the EXO‐D10 have the highest expression among those groups. COL‐1 gene expression of the EXO‐D15 treated cells is much higher than that of the other groups. Runx2 gene expression of the EXO‐D10 treated cells is higher than that of the other groups. These results demonstrate that the EXO‐D10 and EXO‐D15 can induce the osteogenic differentiation of the hMSCs more efficiently than the other exosome samples. Matrix mineralization is a crucial step toward the formation of calcified extracellular matrix associated with the true bone, so Alizarin Red was used to determine the presence of the calcium deposition by the osteogenic lineage. The red deposition in Figure 2A m,n is more obvious than that in Figure 2A k,l,o, further indicating that EXO‐D10 and EXO‐D15 can induce the osteogenic differentiation of the hMSCs more efficiently than the other exosomes. The Alizarin Red intensity (Figure 3e) also showed a similar induction ability of the osteogenic differentiation of the EXO‐D10 and EXO‐D15 exosomes. Meanwhile, the ALP activity was also tested to confirm the osteogenesis of hMSCs. In Figure 3d, EXO‐D10 and EXO‐D15 show a higher ALP activity than the others, consistent with the results of real‐time PCR and immunofluorescence assay. Therefore, EXO‐D10 and EXO‐D15 showed the best ability to induce the osteogenic differentiation of hMSCs due to the significant enhancement in OPN gene and protein expression, calcium deposition, and ALP activity in comparison with the other exosomes.

### 2.3 The Cell‐Free 3D Ti‐Scaffolds with the Exosomes Derived from the Stem Cells Induced the Bone Tissue Regeneration In Vivo

**Figure 4:**
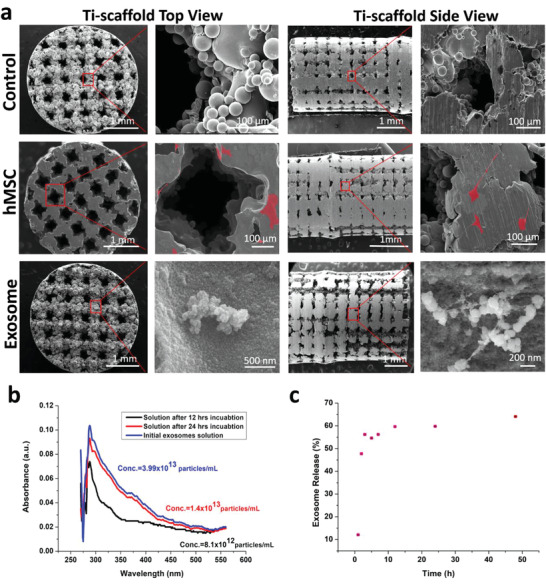
Characterization of the scaffolds and exosome loading into and releasing from the scaffolds. a) SEM images of the Ti‐scaffolds, hMSCs‐Ti‐scaffolds, and exosome‐Ti‐scaffolds. b) Exosomes loading into the Ti‐scaffold and c) exosome releasing from the exosome‐3D‐Ti‐scaffold. (b) shows UV–vis absorption spectra of the initial exosomes solution and the supernatant 12 or 24 h after the exosomes are loaded into the Ti‐scaffold. The exosomes loading efficiency is calculated to be 79.48%. (c) shows the exosome releasing from the Ti‐scaffold in the basal medium (pH = 7.4). hMSCs are pseudo‐colored into red on the hMSCs‐Ti‐scaffolds. The concentration of the exosomes is calculated as the number of exosome particles in per mL.

**Figure 5:**
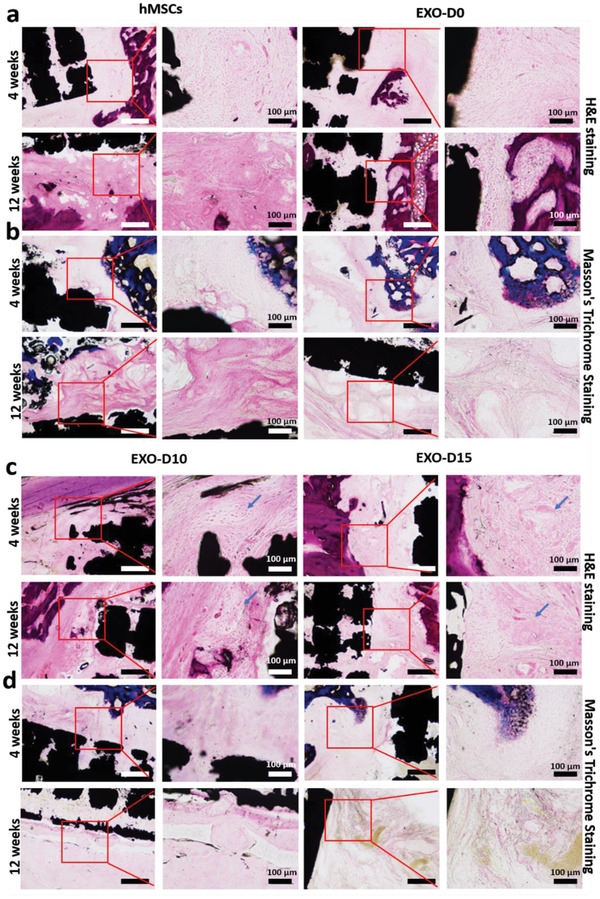
H&E staining and Masson's trichrome staining confirmed the new bone formation in vivo after 4 and 12 weeks. a,c) H&E staining; b,d) Masson's trichrome staining; low magnification scale bar: 250 µm. The arrows indicated the newly formed bone. The black areas reflect the section of the Ti‐scaffolds. The area framed by red square highlights the presence of bone cells. N = 5.

**Figure 6:**
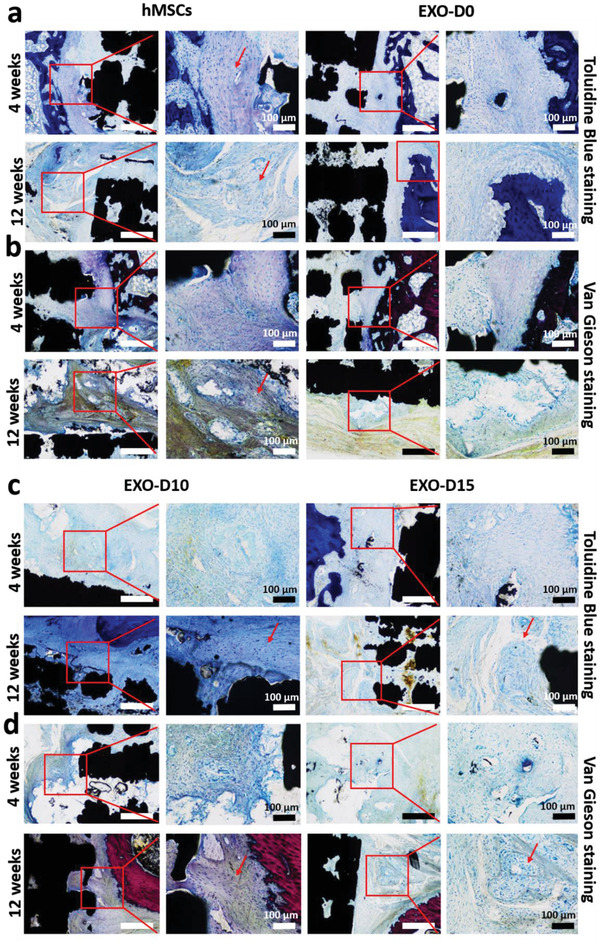
Toluidine Blue staining and Van Gienson staining confirmed the new bone formation in vivo after 4 and 12 weeks. a,c) Toluidine Blue staining; b,d) Van Gienson staining; low magnification scale bar: 250 µm. The arrows indicated the new bone formation. The black areas are the sections of the Ti‐scaffolds. Light pink: the collagen fiber; light blue: the osteoblast cells. *N* = 5.

To demonstrate that the osteogenic exosomes (EXO‐D10 and EXO‐D15) identified by in vitro differentiation assay could enable the cell‐free 3D‐printed Ti‐scaffolds to induce bone regeneration in vivo, we implanted the cell‐free but exosome‐seeded scaffolds (**Figure** **4**) into the rat radial bone defect model to evaluate bone regeneration after 4 and 12 weeks. SEM confirms the porous structures of the cell‐free 3D‐printed scaffolds with and without exosomes (Figure 4). All kinds of exosomes were coated on the 3D‐printed scaffolds, but there is no difference in the exosome coating among those exosome coating scaffolds (data not showed). The exosomes (EXO‐D15) are found to be attached to the surface of the scaffold. We also performed the loading and releasing of the exosomes to the Ti‐scaffold. The exosome loading efficiency after 12 h of incubation is higher than that after the 24 h of incubation and is calculated as 79.48% (Figure 4b). Once loaded, the exosomes are released from the scaffolds continuously and the releasing efficiency can reach up to 50% after 2 h (Figure 4c). These results suggest that the exosomes could be successfully loaded into the scaffolds and released into the surrounding continuously. As a control group, hMSCs‐seeded scaffolds were also prepared (**Figure** **5**) to demonstrate that exosome‐coated cell‐free scaffolds can perform as well as hMSC‐seeded exosome‐free ones in osteogenesis. The newly formed bone tissue and blood vessels were examined by H&E staining, Masson trichrome staining, Toluidine blue staining, and Van Gieson staining (Figures 5 and **6**). The H&E staining assays indicate that the Ti‐scaffolds seeded with the osteogenic exosomes (EXO‐D10 and EXO‐D15) induce the new bone formation, which is evidenced by the presence of new bone cells and Haversian canals like structures (red arrows in Figures 5 and 6), as well as the formation of the blood vessels (blue arrow in Figure S3h, Supporting Information) inside or around the scaffold channels. The collagen production was also confirmed by the three‐color staining (Massion Trichrome Staining, Figure 5b,d). The light green color (collagen protein) was further enhanced in the EXO‐D15 decorated Ti‐scaffolds (Figure 5d) compared to the other EXO‐coated group after 12 weeks of implantation, suggesting that more collagen was produced in the former than in the latter. Meanwhile, osteoblasts (in blue) were also obviously observed both in the hMSCs‐seeded and EXO‐D10, EXO‐D15 groups by the Toluidine blue staining and Van Gieson staining in Figure 6, and we also can find the Haversian canals like structures in Figure 6c,d. It suggested that the EXO‐D10 and EXO‐D15 seeded scaffolds could induce bone tissue regeneration as the hMSCs seeded scaffolds. Overall, the bone formation in the cell‐free scaffolds seeded with the osteogenic exosomes is comparable to that in the hMSCs seeded group 4 and 12 weeks post‐implantation. For the 12 weeks of implantation, the bone tissue regeneration was further enhanced for the EXO‐D15 coated Ti‐scaffolds compared to the EXO‐D10 coated Ti‐scaffolds. In addition, the reduced bone formation was detected in the exosome‐free Ti‐scaffolds (Figure S2, Supporting Information) compared to the exosome‐coated Ti‐scaffolds. Hence, the combination of the 3D Ti‐scaffolds and the osteogenic exosomes could enhance the bone tissue regeneration without externally seeding hMSCs in the scaffolds due to the osteogenesis‐induction capability of exosomes.

It is known that the implantation of biomaterials would cause the inflammatory responses in vivo. Given this, it is likely that the implantation of the scaffolds could influence the body healing by itself at the early stage of the implantation. It is reported that the migration of the inflammatory cells (microphage and giant cells) to the biomaterial's surface may activate the inflammatory response through the production of a wide range of the inflammation involved cytokines in the first two or four weeks.^[^ ^30^ ^]^ In our study, the H&E staining results for 4 weeks post‐implantation show that only a few neutrophils are dispersed in the tissue/scaffold connection area in the hMSCs seeded Ti‐scaffold (Figure S3a,b, Supporting Information). However, no neutrophils were found in the exosome decorated Ti‐scaffold groups (Figure S3c,d,g,h, Supporting Information). This observation indicated that no inflammatory response was caused by the exosomes decorated scaffolds at the early stage of the implantation. Furthermore, we found that there were no inflammatory cells in both the hMSCs‐coated and exosome‐decorated Ti‐scaffold groups 12 weeks post‐implantation (Figure S3e,f, Supporting Information). These results further confirm that the inflammatory response would take place at the early stage of biomaterials implantation.

### 2.4 Mechanism for Osteogenesis of hMSCs Induced by Osteogenic Exosomes

**Figure 7:**
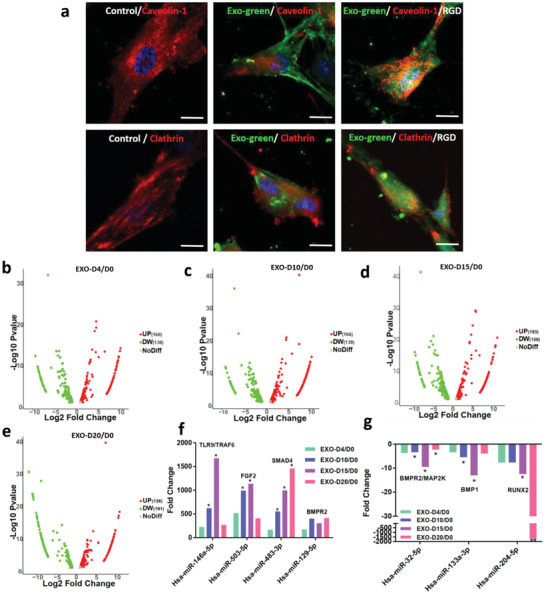
A mechanism for the osteogenesis of hMSCs induced by the osteogenic exosomes. a) Colocalization of the exosomes and clathrin/caveolin‐1 protein. Anti‐caovelin‐1 protein is colocalized with the exosomes labeled with the green fluorescence of the hMSCs and the RGD‐peptide blocked hMSCs as well, but anti‐clathrin protein is not as obvious as the anti‐caveolin‐1. The images are captured by the confocal microscopy. b–e) Volcano analysis for the miRNA expression of different osteogenic exosomes. f) Upregulation and g) downregulation of the miRNAs expression (fold change) involved in the osteogenesis of the hMSCs. FGF: Fibroblast growth factor; BMPR2: Bone Morphogenetic Protein Receptor 2; TRAF6: TNF receptor associated factor; SMAD4: Mothers against decapentaplegic homolog 4; MAP2K: Mitogen‐activated protein kinase kinase; BMP1: Bone morphogenetic protein 1; RUNX2: Runt‐related transcription factor 2. The significant differences are calculated by comparing the EXO‐D4/D0 in each group. Scale bar: 10 µm. *p < 0.05, *N* = 3; **p < 0.01, *N* = 3.

To understand why the two osteogenic exosomes, EXO‐D10 and EXO‐D15, can induce the osteogenic differentiation of the hMSCs in vitro, the exosome endocytosis and next‐generation sequencing (EXONGS) was performed. First, the Exo‐green labeled osteogenic exosomes were incubated with the hMSCs followed by incubation with the Clathrin and Caveolin‐1 antibody. Ideally, the exosomes, as well as Clathrin or Caveolin‐1 protein, should be colocalized because the exosomes are endocytosed by a pathway involved with Clathrin or Caveolin‐1.^[^ ^31^ ^]^ For the green‐labeled exosomes, it is possible that the exosomes were either inside the cells or on the cell surface. The exosomes with a diameter of ≈150 nm were incubated with the hMSCs for about 4 h. During this process, the exosomes might either go into the cells or stay on the surface of the cells (**Figure** **7**a). To confirm if the integrin on the cell membrane and/or exosome membrane is involved, the cell membrane and exosome membrane are blocked with the 2 mM RGD peptide. The high concentration of the RGD peptide could saturate all the integrin receptors on both membranes. From Figure 7a, we can see that the colocalization of the exosome, anti‐caveolin‐1, and anti‐clathrin after the RGD peptide blocking is more obvious than that without blocking. It is further shown that the exosomes are colocalized with the Caveolin‐1 and Clathrin mostly on the cell membrane in contrast to the inside of the cells. However, there are more exosomes colocalized with the Caveolin‐1 (Figure 7a). Therefore, it is most likely that the exosomes go into the cells predominantly by the Caveolin‐1 involved signaling pathway.

**Scheme 2:**
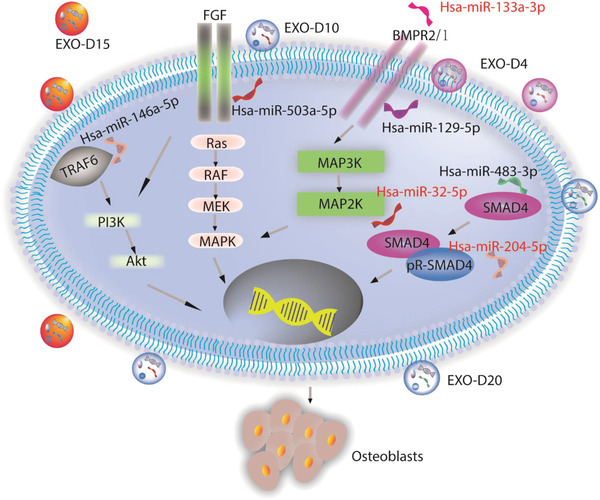
The possible signaling pathways for the exosome‐induced osteogenic differentiation of hMSCs in vitro and in vivo. It is likely that the osteogenic exosomes induce the osteogenesis of the hMSCs by the PI3K/Akt and MAPK signaling pathways. FGF: Fibroblast growth factor; BMPR2: Bone Morphogenetic Protein Receptor 2; TRAF6: TNF receptor associated factor; SMAD4: Mothers against decapentaplegic homolog 4; PI3K: The phosphoinositide 3‐kinase; Akt: serine/threonine‐specific protein kinase; MAPK: microtubule associated protein kinase. MAP2K: Mitogen‐activated protein kinase kinase; BMP1: Bone morphogenetic protein 1.

To evaluate whether the miRNAs in the exosomes are involved in the osteogenesis in vitro, the exosome next‐generation sequencing (NGS) was performed to track the difference in miRNAs in the exosomes among different exosomes samples. Millions of RNAs were detected by the NGS from the commercial service (System Bioscience). The changes in miRNAs expression were shown in Figure 7e–h. Compared to the miRNAs expression in EXO‐D0 exosomes, the expression of 160, 166, 193, and 136 miRNAs were upregulated and that of 130, 139, 150, 191 miRNAs was downregulated in the EXO‐D4, EXO‐D10, EXO‐D15, and EXO‐D20 exosomes, respectively. More importantly, osteogenic miRNAs, such as Hsa‐miR‐146a‐5p,^[^ ^32^, ^33^ ^]^ Hsa‐miR‐503‐5p,^[^ ^34^ ^]^ Hsa‐miR‐483‐3p,^[^ ^35^, ^36^ ^]^ and Hsa‐miR‐129‐5p,^[^ ^37^ ^]^ were upregulated in the EXO‐D10 and EXO‐D15 (Figure 7i). Namely, these miRNAs in EXO‐D10 and EXO‐D15 have a higher abundance than in the other exosomes, which is the possible reason for the enhanced bone tissue regeneration by EXO‐D10 and EXO‐D15 in vitro and in vivo compared to other exosome groups. Meanwhile, some miRNAs, including Hsa‐miR‐32‐5p,^[^ ^38^ ^]^ Hsa‐miR‐133a‐3p,^[^ ^39^ ^]^ and Hsa‐miR‐204‐5p,^[^ ^40^ ^]^ were downregulated in the EXO‐D10 and EXO‐D15 (Figure 7j) obviously. MiRNAs might interact with the growth factors or receptors, such as BMPR2/MAP2K, BMP1, and RUNX2 (according to NGS data analysis, not shown). Hence, the overexpression of pro‐osteogenic miRNAs, such as Hsa‐miR‐146a‐5p, Hsa‐miR‐503‐5p, Hsa‐miR‐483‐3p, and Hsa‐miR‐129‐5p and the downregulation of anti‐osteogenic miRNAs, such as Hsa‐miR‐32‐5p, Hsa‐miR‐133a‐3p, and Hsa‐miR‐204‐5p, were taken together to induce the osteogenic differentiation of the hMSCs. According to the analysis of the NGS sequencing results (Figure S4, Supporting Information), the PI3K/Akt signaling pathway^[^ ^41^ ^]^ and MAPK signaling pathway^[^ ^42^, ^43^ ^]^ (**Scheme** **2**) might play a leading role in the osteogenesis of the hMSCs,^[^ ^44^ ^]^ especially for the osteogenic exosomes of EXO‐D10 and EXO‐D15. MiRNAs might interact with the growth factors or receptors, like TARF6, FGF2, BMPR, BMP1, and RUNX2 (according to NGS data analysis, not shown) to activate the PI3K/Akt and MAPK signaling pathway for the osteogenesis of the hMSCs. Therefore, NGS data explains why two groups of the exosomes, EXO‐D10 and EXO‐D15, can induce the osteogenic differentiation of the hMSCs in vitro and bone tissue regeneration in vivo. Namely, the exosomes, especially EXO‐D10 and EXO‐D15, carry the osteogenic miRNAs to induce the osteogenesis of the hMSCs in vitro and bone tissue regeneration in vivo (Scheme 2).

## 3 Discussion

Small soluble molecules associated with exosomes, which are secreted from the stem cells at the different stages of differentiation, might have different positive effects on the cell differentiation in vitro and tissue regeneration in vivo. Meanwhile, the exosomes can be easily endocytosed into the host cells by the Caveolin‐1 involved signaling pathway (Figure 7d). In our study, we isolated the exosomes from pre‐differentiated stem cells and allowed them to be incubated with the stem cells in basal medium to figure out whether the osteogenic exosomes isolated from different stages could induce the osteogenesis of hMSCs. We found that EXO‐D10 and EXO‐D15 carried osteogenic miRNAs at an upregulated level, such as Hsa‐miR‐146a‐5p, Hsa‐miR‐503‐5p, Hsa‐miR‐483‐3p, Hsa‐miR‐129‐5p (Figure 7i), and the anti‐osteogenic miRNAs (Hsa‐miR‐32‐5p, Hsa‐miR‐133a‐3p, and Hsa‐miR‐204‐5p) at a downregulated level (Figure 7j). Thus, EXO‐D10 and EXO‐D15 can induce the osteogenic differentiation of hMSCs by activating the PI3K/Akt signaling pathway^[^ ^41^ ^]^ and MAPK signaling pathway^[^ ^42^ ^]^ (Figure S3, Supporting Information). Those signaling pathways play important roles in the osteogenesis of the hMSCs and could help to confirm the osteogenic ability of the exosomes. Interestingly, another batch of miRNAs, like miR31,^[^ ^45^ ^]^ miR211,^[^ ^46^ ^]^ and miR‐21^[^ ^47^, ^48^ ^]^ were reported to be negative inducers of osteogenesis and downregulated during the osteogenic differentiation of the MSCs, even though our NGS data did not show those miRNAs. Therefore, the overexpression of pro‐osteogenic miRNAs, such as Hsa‐miR‐146a‐5p, Hsa‐miR‐503‐5p, Hsa‐miR‐483‐3p, and Hsa‐miR‐129‐5p and the inhibition of anti‐osteogenic miRNAs, such as Hsa‐miR‐32‐5p, Hsa‐miR‐133a‐3p, and Hsa‐miR‐204‐5p, acted together to favor the osteogenic differentiation of the hMSCs. This is similar with the earlier finding that some miRNAs could induce the osteogenic differentiation of the hMSCs. In our study, the exosomes from the pre‐differentiated hMSCs, especially those derived from the cells pre‐differentiated for 15 days, could promote the osteogenic differentiation of the hMSCs obviously with the strong ALP activity and accumulation of the mineralization in ECM (Figure 3d). Even though EXO‐D20 also showed a high ALP activity than EXO‐D4, but still lower than EXO‐D10, EXO‐D15 (Figure 3d) in our study. However, a reported study showed that exosomes derived from cells differentiated for 18–21 days were more effective in promoting in vitro osteogenic differentiation.^[^ ^48^ ^]^ Namely, EXO‐D15 in our study showed the best osteogenic ability, which is a little bit different from the reported even later stage (18 and 21 days) exosomes. In a word, the microRNAs profiles in the exosomes derived from the pre‐osteo‐differentiated stem cells can induce the osteogenic differentiation. Furthermore, the osteogenesis of the stem cells was also confirmed by the RT‐PCR, immunofluorescence staining, Alizarin Red Staining, and ALP activity.

The exosomes derived from the stem cells can be easily attached to the poly‐L‐lysine coated 3D Ti‐scaffold (Figure 4). After implantation, the exosomes could release the small molecules, like the miRNAs, to the microenvironments and recruit the cells, then communicate with the surrounding cells by the lipid‐ligand receptor interactions, endocytic uptake, or fusion of vesicles and cell membrane.^[^ ^49^ ^]^ Compared with the exosome‐free Ti‐scaffold implants, the exosome‐coated Ti‐scaffolds showed enhanced bone regeneration, as evidenced by obvious collagen formation and matrix mineralization (Figures 5 and 6). These results show that the osteogenic exosomes we identified can be used to decorate scaffolds to achieve cell‐free bone regeneration. Furthermore, it also showed that this 3D implant has a good osteoconductive property post‐implantation because osteoblast cells and early‐stage bone structures were detected in the cell‐free exosome‐Ti‐Scaffold (Figures 5 and 6). Wen et al. also confirmed that a porous titanium alloy scaffold with a 200–500 µm pore size was biocompatible and osteoconductive, in which the host cells and body fluids could migrate into the porous structure to form new tissues.^[^ ^50^ ^]^ This phenomenon was consistent with our observations. It further showed that this porous cell‐free exosome‐Ti‐scaffold could induce and promote bone tissue regeneration by its specific physical structures and biochemical cues.

In this work, we develop a novel strategy for bone tissue regeneration to treat bone disease. First, the stem cell derived exosomes are used as a bone formation inducer for decorating the 3D‐printed Ti‐scaffolds. Such cell‐free bone tissue regeneration based on the exosomes is a new way to cure bone tissue disease. The cell‐free strategy will not only avoid the immune rejection resulting from the implantation of the foreign cells into the body but also decrease the expense of treating bone defects (Figure S4, Supporting Information). To date, stem cell‐derived exosomes are less immunogenic than the cells because of the low content of membrane proteins, such as major histocompatibility complex (MHC) molecules.^[^ ^17^ ^]^ In a previous study, exosome derived from MSCs were used to co‐incubated with splenic mononuclear cells, which is from an autoimmune encephalomyelitis mouse model. In their study, two interesting phenomena are observed. First, they found that the proliferation of autoreactive lymphocyte was suppressed by adding the exosomes. Second, they discovered that anti‐inflammatory cytokines, including interleukin (IL)‐10 and transforming growth factor (TGF)‐*β* I, increased after incubation of exosomes.^[^ ^51^ ^]^ Based on these studies, we can conclude that exosome can not only avoid the immune response by lack of the MHC complex but also inhibit the immune response by promoting the secretion of anti‐inflammatory factors.

In addition, exosomes derived from the stem cells are successfully used to induce osteogenic differentiation. Achieving such directed differentiation is very important in bone regenerative medicine. Since exosomes can now be isolated using the commercial kits, the use of exosomes is a new and facile approach to directing stem cell differentiation in cell‐free tissue regeneration. To decorate the Ti‐scaffolds with exosomes, polylysine with the positive charge was coated on the surface of the scaffolds for exosome absorption by charge interactions (Figure 4a). Since optimal porosity and high surface/volume could enhance the attachment of soluble molecules and cells, a well‐designed 3D Ti‐scaffold was fabricated by selective laser melting 3D printing^[^ ^52^ ^]^ in our study. A well‐designed void structure could be constructed with higher laser power and lower scanning speed by an improved selective laser sintering method. Also, a gradient temperature was well‐controllable to create an optimal shearing flow for structural fabrication.^[^ ^53^ ^]^ In our work, a 3D porous Ti‐scaffold is well‐fabricated with a 100 W laser power and a slow‐scanning rate (650 mm s^−1^) for exosome loading, cell migration, and attachments. Our 3D Ti‐scaffolds are porous and cylindrical bone‐like structures suitable for load‐bearing bone tissue regeneration. Such scaffolds possess multiple advantageous properties. First, titanium materials are biocompatible and non‐toxic after implantation. Second, Ti‐scaffolds own good mechanical strength to support the bone. Third, Ti‐scaffolds bear 3D structures with an optimal porosity (Figure 4a) for cell migration and proliferation (good conductivity) and have high surface areas for cell attachment. Therefore, by integrating the 3D‐printed Ti‐scaffolds and cell‐derived osteogenic exosomes, this work provides a promising strategy for generating bone tissue in the loading‐bearing sites.

## 4 Conclusion

In conclusion, we isolated exosomes secreted by hMSCs differentiated in the osteogenic medium at different differentiated times. We then added the exosomes into the basal medium to test whether the exosomes could induce the osteogenic differentiation of hMSCs. We found that exosomes secreted by the hMSCs osteogenically pre‐differentiated for 10 and 15 days could most efficiently induce the osteogenic differentiation of hMSCs in basal medium. We finally found that the resultant osteogenic exosomes could be used to decorate the 3D‐printed Ti‐scaffolds to achieve cell‐free bone regeneration within 12 weeks. The exosome‐coated cell‐free Ti‐scaffolds could induce bone regeneration as efficiently as the hMSC‐seeded exosome‐free Ti‐scaffolds. By means of RNA‐sequencing technique, we found that the exosomes could induce the osteogenic differentiation because they contained upregulated osteogenic miRNAs and thus triggered at least two osteogenic differentiation pathways (PI3K/Akt and MAPK). Hence, our study shows that osteogenic exosomes can be identified from pre‐differentiated stem cells and thus used to replace stem cells in tissue regeneration.

## 5 Experimental Section

##### Cell Culture, Exosome Isolation, Identification

The hMSCs were obtained from the Lonza group Ltd. (Lonza, Morristown, NJ, USA). Cells after fewer than five passages were maintained in a humidified atmosphere containing 5% CO_2_ at 37 °C in the Mesenchymal stem cell growth medium Bulletkit (Lonza, Morristown, NJ, USA). hMSCs with about 60% confluency were pre‐differentiated for 4, 10, 15, and 20 days, respectively, by the hMSC Osteogenic BulletKit (Lonza, Morristown, NJ, USA), and exosomes were isolated and termed EXO‐D0 (non‐osteogenic exosome), EXO‐D4, EXO‐D10, EXO‐D15, and EXO‐D20 (osteogenic exosomes), respectively. To isolate exosomes, the osteogenic medium was removed after different days of incubation and cells were washed triple times by PBS. Then, the basal medium (MSCBMTM Basal Media (PT‐3238), Lonza, US) with the 10% exosome‐depleted FBS (EXO‐FBS‐250A‐1, SBI, US) was applied and incubated with the cells for another 24 h at 37 °C and 5% CO_2_. Finally, the above‐conditioned medium was collected for exosome isolation by the ExoQuick‐TC kit (System Bioscience, Palo Alto, CA, USA). The isolated exosomes were resuspended into the 1X PBS. The exosomes were also identified and qualified by the AFM, TEM, NanosightNS300 (Piscataway, NJ, US), and Western Blot.

##### Western Blotting of Exosomes

20 µg of exosome proteins were loaded into the lanes to run sodium dodecyl sulfate polyacrylamide gel electrophoresis. After that, the exosomal proteins were transferred to a polyvinylidene difluoride (PVDF) membrane. The PVDF membrane was blocked with 5% skim‐milk powder and then incubated with different primary antibodies, including exosome specific anti‐CD63 antibody (ab134045, Cambridge, MA, USA), nuclei‐specific anti‐Lamin A+ Lamin C antibody (ab108595, Cambridge, MA, USA), and mitochondria‐specific anti‐TOMM20 antibody (ab78547, Cambridge, MA, USA) and anti‐Cytochrome c antibody (ab90529, Cambridge, MA, USA). Afterward, the PVDF membrane was washed by 1X TBST three times and further incubated with a Goat polyclonal secondary antibody to Rabbit IgG conjugated H&L Alexa Fluor 647 (ab150079, Cambridge, MA, USA), and then imaged by the Bio‐RAD ChemiDoc gel imaging system (Bio‐RAD, Hercules, CA, USA).

##### Exosome Quantification

The isolated exosomes were quantified by the EXOCET Exosome Quantitation kit (System Bioscience, Palo Alto, CA, USA). This kit is based on the activity on Acetyl‐CoA Acetylcholinesterase (AChE),^[^ ^54^, ^55^ ^]^ which is a kind of enzyme enriched in the most kinds of the exosomes. The included calibration standard was able to calculate the standard curve (FS1). It was an easy and quick colorimetric assay. The procedure for the colorimetric assay could be described as follows. First, 20 µL of exosomes were lysed by 80 µL lysis buffer and incubated at 37 °C to liberate exosome proteins. Those proteins were prepared with buffer A and B, provided by the SBI company for the colorimetric assay at 405 nm using a spectrophotometric plate reader.

##### The Osteogenesis of the hMSCs Induced by the Osteogenic Exosomes

hMSCs at 60% confluency were cultured in the hMSCs basal medium (Lonza, Morristown, NJ, USA) in the presence of the osteogenic exosomes (EXO‐D4, EXO‐D10, EXO‐D15, and EXO‐D20) for another 20 days. During this time, 100 µL of the 1.0 × 10^13^ particles per mL osteogenic exosomes were added into the exosome‐free medium and incubated with hMSCs, which were seeded into the 24‐well plates for the induction of the osteogenesis of the hMSCs. The concentration of input exosomes was normalized as ≈10^5^ particles per one cell, which was a high concentration for osteogenic induction in each experimental group. The exosomes were refilled every two days when the medium was changed to avoid the degradation of exosomes.^[^ ^56^ ^]^

##### Immunofluorescence Staining

The hMSCs induced by EXO‐D0, EXO‐D4, EXO‐D10, EXO‐D15, and EXO‐D20, respectively for 20 days were fixed by the 4% PFA at room temperature for 30 min. After the cell membrane was permeabilized by the 0.3% Triton X‐100 for 8 min, osteopontin (OPN) and collagen 1 (COL‐1) were immunostained using the corresponding antibody. After the cells were blocked by the 5% BSA, OPN and COL‐1 were further labeled with the secondary antibody conjugated with the etramethylrhodamine‐5‐isothiocyanate (TRITC) (Abcam, Cambridge, MA, USA). FITC‐labeled phallodin (green) and DAPI (4′,6‐diamidino‐2‐phenylindole) (blue) were used to stain the actin filament and nuclei, respectively. The cells were then imaged by the immunofluorescence microscopy. The quantity of the immunofluorescence staining was calculated by the Image J. The relative fluorescence intensity (RFU) was calculated as follows: RFU = Integrated density − (Area of selected cells × Mean fluorescence of background readings).

##### Real Time PCR

The real‐time PCR was used to analyze the osteo‐specific genes (Alkaline Phosphatase [*ALP*], Runt related transcription factor 2 [*Runx2*], *OPN*) and osteo‐non‐specific genes (*COL‐1*). The Glyceraldehyde 3‐phosphate dehydrogenase (*GAPDH*) was used as a reference gene. Cells were collected on the ice, and real‐time PCR was performed by Ambion Power SYBR Green cells‐to‐Ct Kit (Invitrogen, US). The template cDNA was amplified by genetic primers of *ALP*, *Runx2*, *OPN*, *COL‐1*, and *GAPDH*. The primer sequences were listed as follows. *ALP*: Forward primer: 5′‐CAACCCTGGGGAGGAGAC‐3′, Reverse primer: 5′‐GCATTGGTGTTGTACGTCTTG‐3′;^[^ ^57^ ^]^ *Runx2*: Forward primer: 5′‐CCAGATGGGACTGTGGTTACC‐3′, Reverse primer: 5′‐ACTTGGTGCAGAGTTCAGGG‐3′;^[^ ^58^ ^]^ *OPN*: Forward primer: 5′‐GACGGCCGAGGTGATAGCTT‐3′, Reverse primer: 5′‐CATGGCTGGTCTTCCCGTTGC‐3′;^[^ ^59^ ^]^ *COL‐1*: Forward primer: 5′‐GACGGCCGAGGTGATAGCTT‐3′, Reverse primer: 5′‐CATGGCTGGTCTTCCCGTTGC‐3′;^[^ ^59^ ^]^ *GAPDH*: Forward primer 5′‐ CATGTTCGTCATGGGGTGAACCA‐3′, Reverse primer: 5′‐AGTGATGGCATGGACTGTGGTCAT‐3′.^[^ ^60^ ^]^ The real‐time PCR was performed under the following procedure: initially denatured at 95 °C for 5 min, followed by 45 cycles of PCR (95 °C for 30 s, 58 °C for 30 s, and 72 °C for 45 s).

##### Alkaline Phosphatase Activity and Alizarin Red Staining

Cells were tested for the alkaline phosphatase (ALP) activity and calcium nodule staining (Alizarin red staining) after 20 days of incubation with the osteogenic exosomes (EXO‐D0, EXO‐D4, EXO‐D10, EXO‐D15, and EXO‐D20), respectively. ALP activity was evaluated by the *ρ*‐Nitrophenyl phosphate (pNPP) assay. Briefly, pNPP (Invitrogen, Waltham, MA, USA) was used as a substrate for the ALP activity analysis where the ALP was hydrolyzed to form a soluble yellow solution at 37 °C. The staining reaction was terminated by adding 3 m NaOH and the absorbance was read at 405 nm. For the alizarin red staining, cells were first fixed by the 4% PFA at 4 °C for 15 min and stained with 0.2% alizarin red for 15 min. The staining images were captured by the optical microscope.

##### Colocalization of the Exosomes and Clathrin or Caveolin‐1 Membrane Protein

The hMSCs were incubated with the osteogenic exosomes labeled with the Exo‐Green (System Bioscience, Palo Alto, CA, USA) for 4 h. Meanwhile, the 2 mm RGD‐peptide blocked hMSCs and exosomes‐Green were also incubated together for 4 h. Cells were rinsed and fixed by 4% PFA for 30 min. They were subsequently permeabilized using 0.3% Triton X‐100 for 8 min, followed by blocking with the 5% BSA for 1 h. After blocking, the clathrin (Abcam) and caveolin‐1 (Abcam) antibody were applied to the fixed cell at 4 °C overnight, respectively. The secondary antibody conjugated with the TRITC was used for labeling the clathrin and caveolin‐1 for 1 h. The images were captured using confocal microscopy (Leica SP8 Upright CLS/multiphoton/FLIM microscope, Rockland, MA, USA).

##### Exosome Next‐Generation Sequencing

The exosomes were isolated from the different culture mediums (hMSCs pre‐differentiated for 0, 4, 10, 15, and 20 days, respectively) by following the commercial protocol by the System Bioscience. Then the next generation sequencing (System Bioscience, Palo Alto, CA, USA) was applied to track the miRNA expression. The raw fastq data quality was checked by the FASTQC, meanwhile, the adaptor was removed to trim quality bases by the Trimmomatic. The leading and trailing ambiguous or low‐quality bases, which were below Phred quality scores of 3, were also removed after adapter clipping. The miRNA read counting was detected by the Chimirra and the miRNA expressions were normalized by the trimmed mean of M‐values (TMM). The edgeR program was further used to identify the differentially expressed genes. The gene with a fold change of expression more than 1.5 was defined as a differentially expressed one. The miRNA target gene prediction was detected by the IPA and the clusterProfiler R was also performed to conduct the GO and pathway enrichment analysis.

##### Construction of the Osteogenic 3D Ti‐Scaffold

The Ti‐scaffolds (8 mm in length, and 3 mm in diameter), 3D‐printed from Ti6Al4V powder, were first autoclaved and then cooled to the room temperature. The poly‐L‐lysine was coated on the 3D Ti‐scaffolds by incubating the scaffolds in a solution of poly‐L‐lysine overnight before exosome coating. The exosomes (EXO‐D0, EXO‐D4, EXO‐D10, and EXO‐D15) were seeded onto the Ti‐scaffold overnight at 4 °C. The osteogenic exosomes attached on the surface were confirmed by the SEM (Zeiss Neon 40 EsB, NY, USA).

##### The Exosome Loading and Releasing Behaviors of the 3D Ti‐Scaffold

3.99 × 10^9^ particles per mL exosomes were incubated with the poly‐L‐lysine coated 3D Ti‐scaffolds for 12 and 24 h, respectively, at 4 °C. The loading efficiency% was calculated as follows: loading efficiency% = the number of the exosomes loaded to the 3D Ti‐scaffolds/the number of initial exosomes × 100%. The number of the loaded exosomes was equal to the difference between the number of initial exosomes the number of unloaded exosomes. The exosomes releasing from the 3D Ti‐scaffolds were performed at the basal medium, pH = 7.4, 37 °C at 1, 2, 3, 5, 7, 12, 24, and 48 h. The exosome releasing efficiency was determined using the equation below: The exosome releasing efficiency% = the number of exosomes in the supernatant/number of exosomes loaded into the 3D Ti‐scaffolds × 100%.

##### *In Vivo* Evaluation of the Osteogenic 3D Ti‐Scaffold

The in vivo study was approved by the Institutional Animal Care and Use Committee of the University of Oklahoma (R17‐028). The male 5–6 weeks old Sprague Dawley (SD) rats (Harlan, ≈125 g) were randomly separated into 7 groups (*n* = 5), including healthy group, negative group, EXO‐D0, EXO‐D4, EXO‐D10, EXO‐D15, and hMSCs cell‐seeded group. The SD rats were anesthetized with the isoflurane, and a segmental defect about 8 mm was created. The exosome seeded 3D Ti‐scaffold and its positive/negative control was loaded into the defect zone. The blank control was also created without loading any implantation. The animals were sacrificed to evaluate the bone tissue regeneration using the hematoxylin and eosin (H&E), Masson's Trichrome, Toluidine blue, and Van Gieson staining. The osteogenic 3D‐Ti scaffolds were collected from the rats 4 and 12 weeks post‐implantation, respectively, put into 10% fresh formalin solution, and immersed for 1 day. The fixed 3D‐Ti scaffolds were decalcified first, followed by encapsulation with a paraffin block. Subsequently, the implanted scaffolds were sectioned into slices (4 µm thick) using Histology Cyro Dry Diamond knife on a rotatory microtome (RM2016, Lecia, Germany). Before the scaffolds were sectioned, the sample holder was set up with the advancement of the same thickness (4 µm) at the rotary motion's highest point. A fresh section was then produced by cutting and kept on the diamond knife through the forward motion of the sample holder. The next section continued to be cut by a rotary microtome. The cutting speed was manually controlled because Ti‐scaffolds were too hard to cut. The sections were polished until they were thin enough for microscopy imaging and placed onto the glass slides. After deparaffinated, these slices were stained with H&E, Masson's Trichrome, Toluidine blue, and Van Gieson, and then imaged.

##### Statistical Analysis

The data were presented as the mean ± standard deviation. They were analyzed by the one‐way ANOVA with Tukey's post‐hoc test using SPSS21 (IBM, USA) with *p* < 0.05 showing the significant difference.

## Conflict of Interest

The authors declare no conflict of interest.

## Supporting information

Supporting Information
