# Supplementary material for: Human Mesenchymal Stem Cell Derived Exosomes Enhance Cell‐Free Bone Regeneration by Altering Their miRNAs Profiles
Source: Adv Sci (Weinh). 2020 Aug 7;7(19):2001334. doi: 10.1002/advs.202001334 (PMC7539212; doi:10.1002/advs.202001334)
Supplement: Supplementary file 1 — Supporting Information [file ADVS-7-2001334-s001.pdf]

© 2020 Wiley-VCH GmbH

ADVANCED SCIENCE
Open Access

Supporting Information

for *Adv. Sci.*, DOI: 10.1002/advs.202001334

Human Mesenchymal Stem Cell Derived Exosomes Enhance Cell-Free Bone Regeneration by Altering Their miRNAs Profiles

*Mengmeng Zhai, Ye Zhu, Mingying Yang, and Chuanbin Mao**

Supplementary information

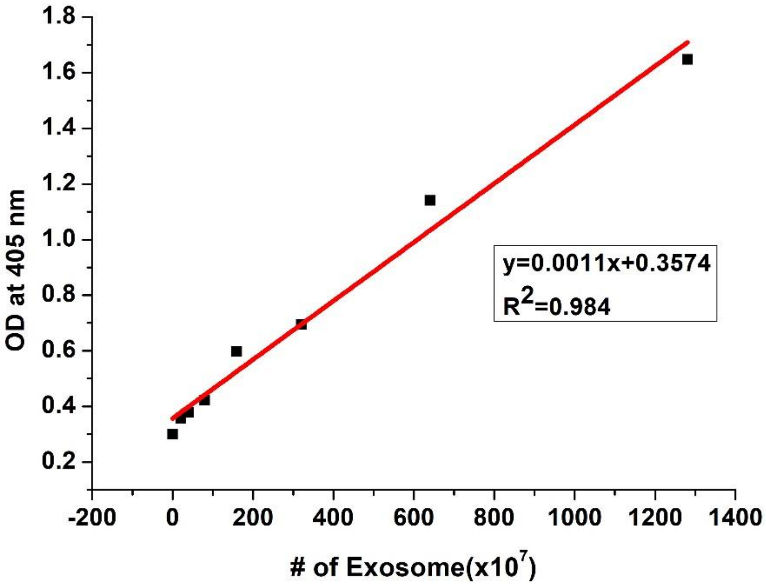

Figure S1. Standard curve of the exosome samples.

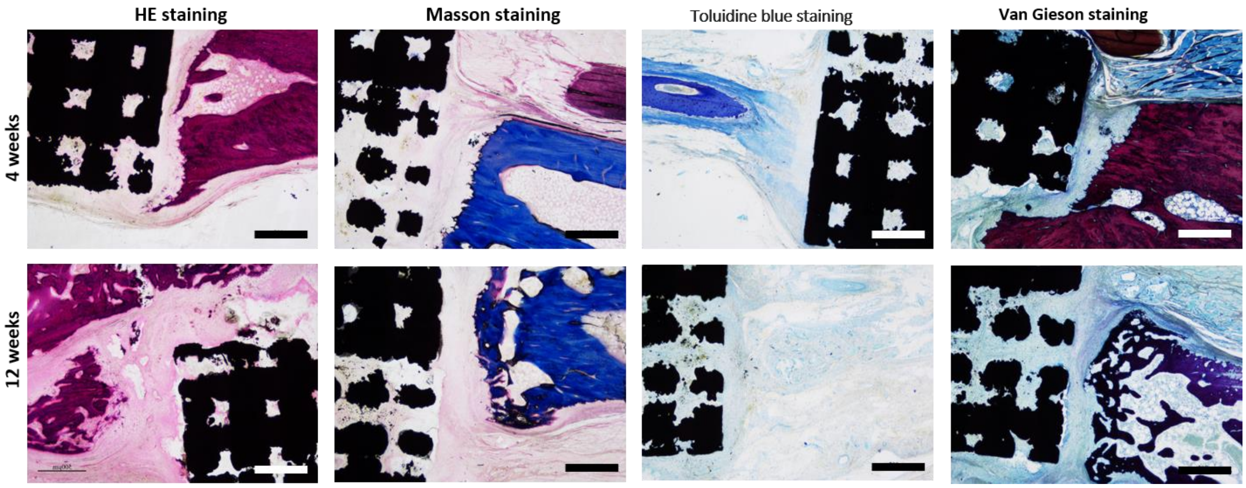

Figure S2. Reduced bone tissue regeneration formation in the channels of Ti-scaffolds 4 and 12 weeks post implantation, respectively. Scale bar: 500 μm.

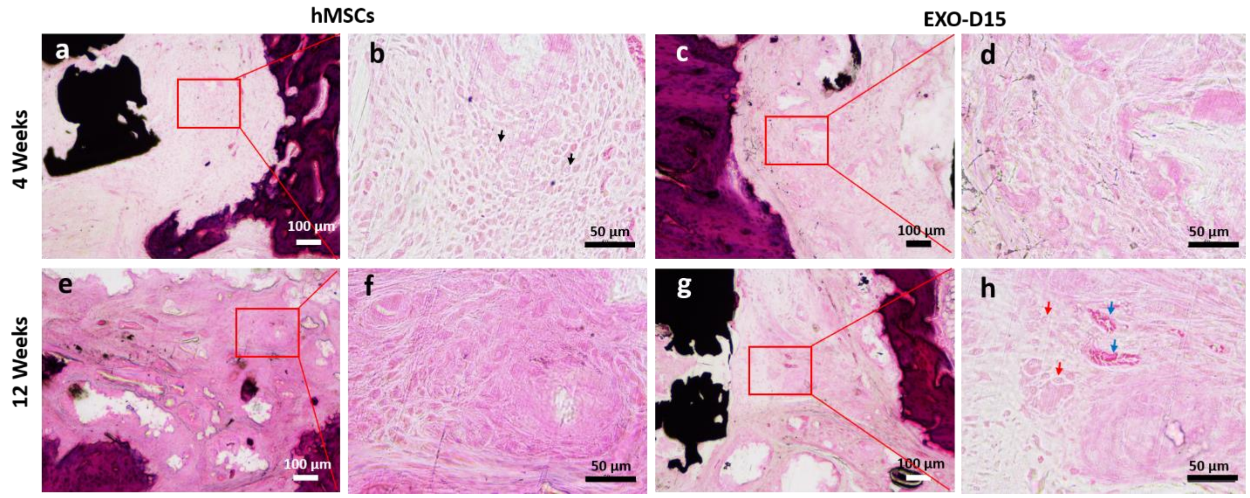

Figure S3. Histological analysis of the hMSCs decorated Ti-scaffolds (a, b, e and f) and EXO-D15 decorated Ti-scaffolds (c, d, g and h). The black arrows show that the neutrophils were dispersed in the boundary between the tissue and Ti-scaffold 4 weeks post implantation but were not present 12 weeks post implantation, suggesting some immune reaction after 4 weeks of implantation (a and b) and no obvious immune response after 12 weeks of implantation (e and f) in the hMSCs decorated Ti-scaffolds. No obvious inflammatory response was found in the EXO-D15 decorated Ti-scaffolds after both 4 weeks (c and d) and 12 weeks (g and h) of implantation, suggesting the absence of immune reaction in EXO-D15 decorated Ti-scaffolds. Red arrows: osteoblasts. Blue arrows: blood vessels.

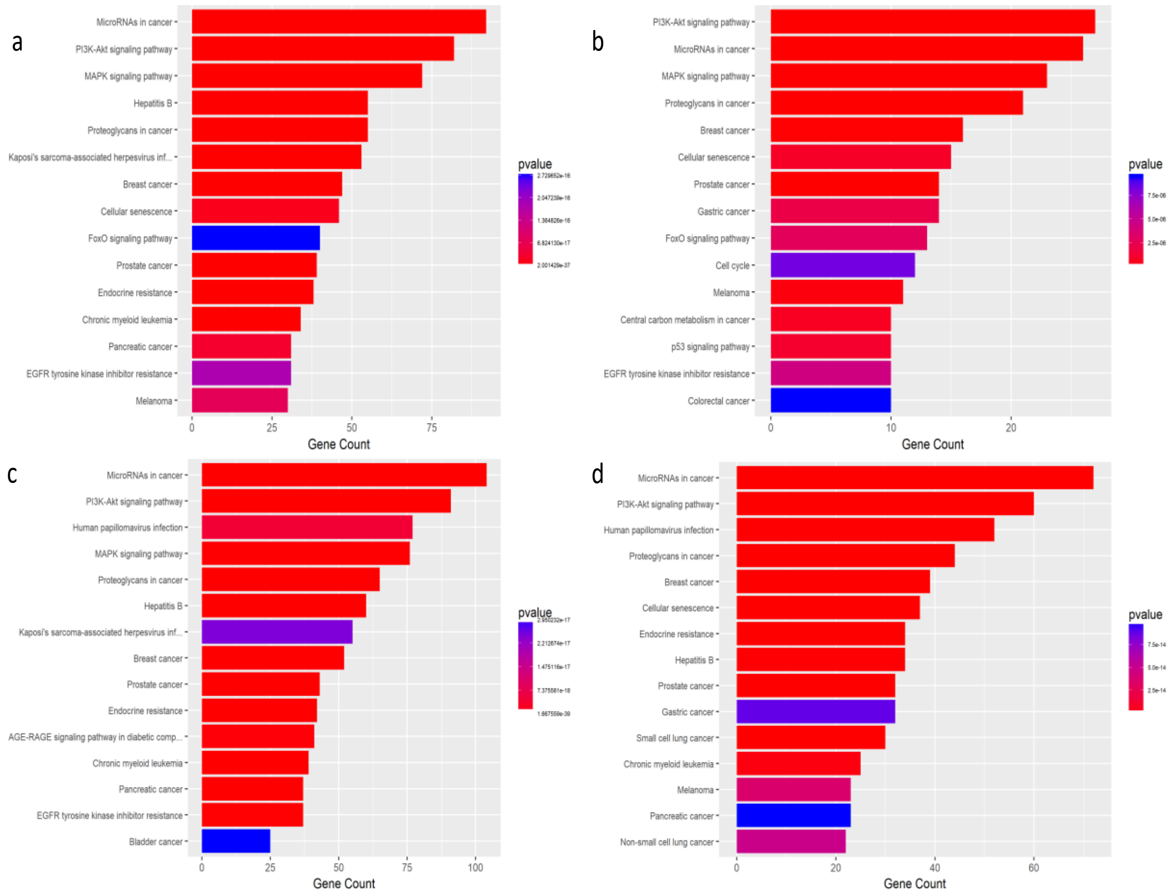

Figure S4. The possible signaling pathways under the mediation of osteogenic exosomes derived from stem cells pre-differentiated for 4 (a), 10 (b), 15 (c) and 20 (d) days.
